# MASE-GC: a multi-omics autoencoder and stacking ensemble framework for gastric cancer classification

**DOI:** 10.3389/fcell.2025.1704237

**Published:** 2025-11-12

**Authors:** Di Liu, Zhongguang Che, Guannan Xu, Ye Huang

**Affiliations:** First Affiliated Hospital of Jinzhou Medical University, Jinzhou, China

**Keywords:** gastric cancer, multi-omics, autoencoder, ensemble learning, XGBoost, oncology

## Abstract

**Background:**

Gastric cancer (GC) is one of the most common malignant tumors and remains a leading cause of cancer-related mortality worldwide. Accurate classification of GC is critical for improving diagnosis, prognosis, and personalized treatment. Recent advances in high-throughput sequencing have enabled the generation of large-scale multi-omics data, offering new opportunities for precise disease stratification. However, existing studies often rely on single-omics approaches or single-model frameworks, which fail to capture the full complexity of tumor biology and suffer from limited sensitivity, specificity, and generalizability.

**Methods:**

We propose MASE-GC (Multi-Omics Autoencoder and Stacking Ensemble for Gastric Cancer), a novel computational framework that integrates exon expression, mRNA expression, miRNA expression, and DNA methylation profiles. MASE-GC employs modality-specific autoencoders to extract compact latent features from heterogeneous omics layers and combines them through weighted fusion. The integrated features are then classified using a stacking ensemble of five base learners—Support Vector Machine, Random Forest, Decision Tree, AdaBoost, and Convolutional Neural Network—followed by an XGBoost meta-classifier. A robust preprocessing pipeline, including feature filtering, normalization, and SMOTE–Tomek balancing, is incorporated to address noise, high dimensionality, and class imbalance.

**Results:**

Comprehensive experiments on the TCGA-STAD cohort demonstrated that MASE-GC achieved superior classification performance compared with single-omics and baseline methods, reaching an accuracy of 0.981, precision of 0.9845, recall of 0.992, F1-score of 0.9883, and specificity of 0.824. Ablation studies confirmed the complementary contributions of autoencoders and ensemble components, with CNN and Random Forest providing the largest performance gains. Furthermore, independent validation on external cohorts (GSE62254, GSE15459, GSE84437, and ICGC) confirmed the robustness and generalizability of MASE-GC, with accuracy consistently above 0.958 and F1-scores exceeding 0.969.

**Conclusion:**

MASE-GC advances computational oncology by offering an effective and generalizable framework for GC classification. By integrating multi-omics fusion, ensemble learning, and robust preprocessing, the proposed model improves both sensitivity and specificity, reduces false positives, and demonstrates strong potential for clinical translation in precision diagnostics and treatment planning.

## 1 Introduction

Gastric cancer (GC) remains one of the most prevalent malignancies worldwide and a leading cause of cancer-related mortality (Sung et al., 2021; The Cancer Genome Atlas Research Network, 2014). Despite advances in diagnosis and treatment, prognosis for many GC patients remains poor, primarily due to tumor heterogeneity, late-stage detection, and limited sensitivity of traditional biomarkers. Recent progress in high-throughput sequencing technologies has enabled the generation of large-scale multi-omics datasets—including exon expression, mRNA expression, miRNA expression, and DNA methylation—that provide complementary molecular insights into cancer initiation and progression (Cristescu et al., 2015; Goldman et al., 2020; International Cancer Genome Consortium, 2010). These data offer unique opportunities for improving classification and risk stratification of GC patients, yet fully exploiting them poses significant methodological challenges.

In recent years, machine learning and deep learning techniques have been widely applied to cancer classification (Zou et al., 2019; Angermue et al., 2016). Classical machine learning models, such as support vector machines (SVMs), decision trees (DTs), random forests (RFs), and ensemble boosting methods, have shown strong predictive ability in handling structured omics datasets (Cortes and Vapnik, 1995; Breiman, 2001; Freund et al., 1997). Meanwhile, deep learning architectures such as convolutional neural networks (CNNs), recurrent neural networks, and graph neural networks (GNNs) have demonstrated superior capacity to capture nonlinear patterns and high-order interactions in large-scale biological data (Zhou and Troyanskaya, 2015; Kipf and Max, 2017). These approaches have yielded promising results across various cancer types, including GC.

Nevertheless, several limitations persist. First, the majority of studies rely on single-omics data, for instance, using only mRNA expression or DNA methylation (Huang et al., 2017; Subramanian et al., 2020). While these approaches can reveal partial molecular mechanisms, they inevitably overlook the complementary and cross-regulatory relationships among different omics layers. As a result, models built on single-omics inputs often suffer from reduced sensitivity and specificity, limiting their translational value. Second, single-model frameworks are prone to overfitting and struggle with high-dimensionality, data imbalance, and noise—factors that are common in biomedical datasets. For example, CNNs can capture complex dependencies but require large sample sizes, while traditional classifiers such as RF and SVM may fail to generalize across heterogeneous cohorts. Third, existing integration attempts, including simple feature concatenation or early/late fusion strategies, have shown improvements but often lack robustness and interpretability when applied to independent validation cohorts.

Recent works have begun to explore multi-omics integration to address these challenges (Wang et al., 2014; Shen et al., 2009; Argelaguet et al., 2018). Multi-omics approaches exploit the complementary nature of different biological layers, offering improved classification accuracy and biological interpretability compared to single-omics methods. However, current integration strategies often face difficulties in dimensionality reduction, noise elimination, and balanced learning (Duan et al., 2021; Heo et al., 2021). Moreover, many studies do not employ ensemble architectures that can fully leverage the complementary strengths of diverse classifiers, thereby limiting their overall predictive stability.

The objective of this study is to develop a robust and generalizable computational framework for GC classification through comprehensive multi-omics integration. To this end, we propose MASE-GC (Multi-Omics Autoencoder and Stacking Ensemble for GC), which combines feature extraction via modality-specific autoencoders with classification through a stacking ensemble of diverse base learners. By simultaneously exploiting linear, nonlinear, and deep learning paradigms, MASE-GC is designed to enhance predictive performance while maintaining interpretability and clinical applicability.

The main contributions of this work can be summarized in three aspects:

1. Multi-omics integration with autoencoders: We introduce a modality-specific autoencoder strategy to extract compact latent features from exon, mRNA, miRNA, and DNA methylation data, enabling effective dimensionality reduction and noise minimization across heterogeneous omics layers.
2. Stacking ensemble architecture: We design a novel ensemble that integrates SVM, RF, DT, AdaBoost, and CNN as base learners, with an XGBoost meta-classifier to combine their complementary strengths for improved sensitivity and specificity.
3. Robust preprocessing pipeline: We implement a systematic preprocessing workflow including normalization, feature selection, and hybrid Synthetic Minority Oversampling Technique (SMOTE)-Tomek resampling, ensuring reliable learning in the presence of high-dimensionality, imbalance, and noisy biological data.

Through extensive experiments on both internal and external GC cohorts, we demonstrate that MASE-GC significantly outperforms single-omics and baseline approaches, offering a promising pathway toward precision oncology in GC classification.

Unlike other multi-omics frameworks such as CRIA and CMIM, which primarily focus on single-model approaches or limited multi-omics integrations, MASE-GC uniquely combines modality-specific autoencoders for feature extraction with a diverse stacking ensemble of classifiers, offering enhanced predictive performance, robustness, and generalizability across multiple datasets.

## 2 Data

### 2.1 Dataset

In this study, we collected four types of omics profiles from the TCGA stomach adenocarcinoma (TCGA-STAD) cohort: exon expression, mRNA expression, mature miRNA expression, and DNA methylation (Zhang et al., 2014). All datasets were retrieved from the UCSC Xena repository (https://xenabrowser.net/datapages/). Exon and mRNA expression profiles were generated using the Illumina HiSeq 2000 sequencing platform, with exon data quantified as RPKM (Reads Per Kilobase per Million mapped reads) (Mortazavi et al., 2008). DNA methylation was measured using the Illumina Infinium HumanMethylation450 array and expressed as beta values, while mature miRNA levels were obtained through Illumina HiSeq small RNA sequencing (Bibikova et al., 2011). The sample distribution and feature dimensions of the original datasets are summarized in Table 1.

**TABLE 1:** Summary of the original multi-omics datasets.

Dataset	SC samples	Normal samples	Number of features	Platform
Exon expression	415	35	239,322	NGS
mRNA expression	415	35	20,530	NGS
miRNA expression	387	41	2,178	NGS
DNA methylation	396	29	48,577	Microarray

### 2.2 Dataset preprocessing

To ensure data quality and comparability across omics layers, we conducted a multi-step preprocessing workflow. First, low-abundance features were filtered out; features with zero counts in more than 60% of samples were excluded. For DNA methylation, missing values were imputed using K-nearest neighbor (KNN) interpolation (Troyanskaya et al., 2001). Next, all retained features were normalized via min–max scaling to the [0, 1] range, preserving relative differences while harmonizing measurement scales across sequencing and array platforms. After normalization, differential feature screening was performed using the LIMMA R package, which supports both RNA-Seq and microarray datasets through the voom transformation (Law et al., 2014; Ritchie et al., 2015). Statistically relevant features were identified under a Benjamini–Hochberg adjusted P-value threshold of <0.001 (Benjamini and Yosef, 1995). Finally, since the downstream analysis required joint representation, the preprocessed omics profiles were aligned at the sample level, retaining only those subjects with complete measurements across all four omics layers.

### 2.3 Dataset balancing

As shown in Table 2, the raw datasets exhibited a severe imbalance between SC and normal samples. To mitigate bias introduced by this imbalance, we applied a hybrid resampling strategy combining SMOTE with Tomek link undersampling (Chawla et al., 2002; Jia et al., 2021; Batista et al., 2004). SMOTE was employed to generate synthetic instances of the minority class (normal tissue samples), while Tomek links were used to eliminate ambiguous samples lying near class boundaries, thereby reducing noise and overlap. This approach not only improved class distribution but also provided a cleaner dataset for model training. Following preprocessing, feature filtering, and sample alignment across multiple omics layers, the resampled balanced dataset is summarized in Table 2, which includes the integrated multi-omics dataset with matched SC and normal cohorts.

**TABLE 2:** Summary of the aligned and balanced multi-omics datasets after applying the SMOTE-Tomek algorithm.

Dataset	SC samples	Normal samples	Number of features
Exon expression	415	415	239,322
mRNA expression	415	359	20,530
miRNA expression	387	438	2,178
DNA methylation	396	396	48,577
Integrated multi-omics	367	367	658,086

## 3 Methods

**FIGURE 1:**
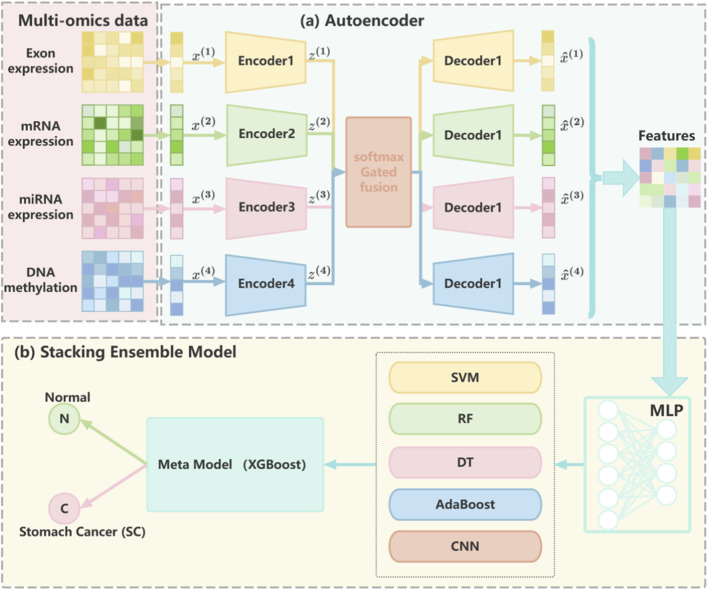
The overall architecture of MASE-GC, consisting of **(a)** modality-specific autoencoders for multi-omics feature extraction and **(b)** a stacking ensemble classifier with five base learners and an XGBoost meta-model for GC classification.

In this study, we propose MASE-GC (Multi-Omics Autoencoder and Stacking Ensemble for GC), a cancer classification framework that integrates multi-omics profiles and a stacking ensemble strategy to improve prediction performance in GC. The overall workflow of MASE-GC is illustrated in Figure 1, which contains two main components: (a) feature extraction via multi-layer autoencoders and (b) classification through a stacking ensemble model.

First, four omics modalities—exon expression, mRNA expression, miRNA expression, and DNA methylation—are separately processed by modality-specific autoencoders. Each autoencoder compresses the high-dimensional input into a latent representation, thereby reducing dimensionality and capturing informative biological patterns while minimizing noise and redundancy (Hinton and Ruslan, 2006; Vincent et al., 2008). The latent codes from the four omics sources are then integrated through a weighted fusion mechanism to form a unified representation for each patient (Figure 1A).

Second, the integrated features are fed into the stacking ensemble classifier of MASE-GC. Five diverse base learners—Support Vector Machine (SVM), Random Forest (RF), Decision Tree (DT), Adaptive Boosting (AdaBoost), and Convolutional Neural Network (CNN)—are employed to capture complementary strengths across linear, nonlinear, and deep learning paradigms. The prediction probabilities from these base models are concatenated and used as inputs to a meta-model implemented with XGBoost, which learns correlations among base predictions to refine the final classification (Figure 1B) (Wolpert, 1992; Chen and Guestrin, 2016).

### 3.1 Multi-omics feature extraction via autoencoder

To effectively handle the high dimensionality and heterogeneity of multi-omics data, we adopt a multi-layer autoencoder framework to learn compact latent representations from four omics modalities, namely, exon expression, mRNA expression, miRNA expression, and DNA methylation. Each modality is first encoded into a low-dimensional representation, and then reconstructed through its corresponding decoder to preserve informative features while minimizing redundancy.

The encoder for the *i*th sample at the *l*th layer is defined as (see Equation 3.1):

$$Z_{i}^{\left(l\right)}=f_{e}\left(Z_{i}^{\left(l-1\right)}\right)=\sigma \left(W_{i}^{\left(l\right)T}\;Z_{i}^{\left(l-1\right)}+b_{i}^{\left(l\right)}\right)$$ (3.1)

where $Z_{i}^{\left(0\right)}=X_{i}$ denotes the original omics features, $W_{i}^{\left(l\right)}$ and $b_{i}^{\left(l\right)}$ represent the weight matrix and bias at layer lll, and $\sigma \left(\cdot \right)$ is the activation function.

The decoder reconstructs the original data from the latent code, expressed as (See Equation 3.2):

$${\tilde{X}}_{i}^{\left(l\right)}=f_{d}\left(Z_{i}^{\left(l\right)}\right)=\sigma \left({\left(W_{i}^{\left(l\right)}\right)}^{T}Z_{i}^{\left(l\right)}+{{b_{i}}^{\prime }}^{\left(l\right)}\right)$$ (3.2)

where ${\tilde{X}}_{i}^{\left(l\right)}$ represents the reconstructed features of sample *i*.

To measure the reconstruction quality, the Mean Squared Error (MSE) loss is applied (See Equation 3.3):

$$L_{\text{MSE}}\left(X_{i},{\tilde{X}}_{i}^{\left(L\right)}\right)=\frac{1}{n}{\sum_{i=1}^{n}\left\|X_{i}-{\tilde{X}}_{i}^{\left(L\right)}\right\|}_{2}^{2}$$ (3.3)

where *n* is the number of samples and *L* is the number of layers

$$L_{\text{AE}}=\sum_{i=1}^{M}\lambda_{i}\;L_{\text{MSE}}\left(X_{i},{\tilde{X}}_{i}^{\left(L\right)}\right)$$ (3.4)

where M=4 is the number of modalities, $\lambda_{i}\geq 0$ is the weight assigned to modality *i*, and $\sum_{i=1}^{M}\lambda_{i}=1.$

Finally, a unified latent representation is obtained by integrating the modality-specific latent codes with the same set of weights (See Equation 3.5):

$$Z=\sum_{i=1}^{M}\lambda_{i}\;Z_{i}^{\left(L\right)}$$ (3.5)

where *Z* represents the shared feature space that captures complementary information from exon, mRNA, miRNA, and DNA methylation profiles. This latent embedding is subsequently used for downstream predictive modeling.

### 3.2 Stacking ensemble model

To improve predictive accuracy and stability in multi-omics cancer classification, we designed a stacking framework that incorporates five complementary base learners—SVM, RF, DT, AdaBoost, and CNN—followed by a meta-classifier that integrates their predictions. These models were selected because each provides unique strengths in handling nonlinearity, high-dimensionality, data imbalance, and noise, which are all common in biological datasets.

#### 3.2.1 Support vector machine (SVM)

SVM constructs separating hyperplanes in a transformed feature space, with the objective of maximizing the margin between classes. By employing kernel functions, it effectively addresses nonlinear separability and high-dimensional data challenges. In cancer genomics, SVM is particularly advantageous because omics datasets often contain far more features than samples. Its strong theoretical foundation and resistance to overfitting with proper regularization make it a reliable choice. We included SVM to capture subtle decision boundaries in the selected omics features.

#### 3.2.2 Random forest (RF)

RF is an ensemble of decision trees built using bootstrap sampling and random feature selection. This diversity among trees reduces overfitting and improves generalization. It is especially robust against noisy features and redundant variables, both of which are prevalent in large-scale omics data. Compared with a single tree, RF balances bias and variance effectively, and its ability to measure feature importance provides interpretability. RF was selected for its stability and proven performance in high-dimensional biomedical classification tasks.

#### 3.2.3 Decision tree (DT)

DTs provide a transparent tree-like structure where internal nodes represent decisions on feature values, branches denote outcomes, and leaves correspond to class labels. Their major strengths are simplicity, intuitive interpretability, and efficiency in handling categorical or discrete features. Although they are prone to overfitting and sensitive to outliers when used alone, their inclusion in stacking adds diversity to the ensemble. DTs were chosen to complement more complex models by providing straightforward decision rules that may capture clinically meaningful thresholds in molecular data.

#### 3.2.4 Adaptive boosting (AdaBoost)

AdaBoost is a boosting technique that sequentially trains weak learners, often decision trees, and assigns higher weights to misclassified samples in subsequent iterations. This iterative reweighting enables the model to focus on difficult cases, improving performance on imbalanced datasets—a frequent scenario in cancer studies where normal samples are often fewer than tumor samples. AdaBoost is sensitive to noise but excels in refining boundaries where other classifiers may fail. We selected AdaBoost for its adaptive nature and ability to highlight minority-class signals.

#### 3.2.5 Convolutional neural network (CNN)

CNNs, though originally developed for image analysis, have been successfully adapted to genomics and transcriptomics data. By applying one-dimensional convolutional filters, CNNs capture local patterns and interactions across ordered features, automatically extracting hierarchical representations (Kelley et al., 2016). This capability is crucial in omics integration, where relationships between adjacent genomic elements or expression features can provide predictive signals. CNN was chosen as a base learner for its strength in uncovering complex nonlinear dependencies that traditional classifiers might overlook.

#### 3.2.6 Meta-model

The prediction probabilities from the five base models are concatenated to form a new feature set, which is then passed to a higher-level learner (XGBoost). This meta-model captures correlations among the outputs of base classifiers and learns to correct their systematic errors. By combining SVM’s margin-based separation, RF’s robustness, DT’s interpretability, AdaBoost’s adaptive focus, and CNN’s deep feature extraction, the stacking architecture achieves a more balanced and powerful classification system than any individual model.

### 3.3 MASE-GC algorithm implementation

In the MASE-GC framework, we assume the feature dimension of each omics dataset is high, and autoencoders are adopted to reduce dimensionality and capture compact latent features. For base classifiers, we determine optimal hyperparameters through grid search. Specifically, for SVM we use the RBF kernel with $C=\left\{0.001,0.01,0.1\right\}$ and $\gamma =\left\{1,10,100\right\}$ ; for Random Forest we set $n\_estimators=\left\{200,500\right\}$ ; for Decision Tree, default parameters are applied; for AdaBoost we set $n\_estimators=\left\{100,200,300\right\}$ ; for CNN, kernel size, filter number, and dropout are tuned empirically. The meta-classifier XGBoost is used with n_estimators=50 . Let *h* denote a base learner, $L_{f}$ the training set of the *f*th fold, ${Test}_{f}$ the corresponding test set, and $T_{fk}$ the *k*th sub-training set within fold *f*. The pseudocode of MASE-GC is summarized in Algorithm 1.

Algorithm 1 MASE-GC.

**Input: Multi-omics dataset *D*, labels** $Y\in R^{m}$

**Output:** Prediction results
**Step 1: Feature Extraction via Autoencoders**
(1) Apply 10-fold cross-validation, splitting *D* into training set $L_{f}$ and test set ${Test}_{f}$ , *f = 1, … ,10.*
(2) Train modality-specific autoencoders (Exon, mRNA, miRNA, DNA methylation) on $L_{f}$ to obtain latent features $Z^{\left(k\right)}$ .
(3) Fuse latent features across modalities with weighted integration to construct a unified representation *Z.*
**Step 2: Train Stacking Ensemble Model**
(4) Apply 10-fold cross-validation, splitting $L_{f}$ into training set $T_{fk}$ and validation set $V_{fk}$ , *k = 1, … ,10.*
(5) a. Train five base classifiers—SVM, RF, DT, AdaBoost, CNN—on $T_{fk}$ .
(5) b. Optimize hyperparameters of each base model using grid search on $V_{fk}$ .
(5) c.Construct a new dataset $\left\{x_{i}^{\prime },y_{i}\right\}$ , where $x_{i}^{\prime }=\left\{h_{1}\left(x_{i}\right),h_{2}\left(x_{i}\right),\ldots ,h_{5}\left(x_{i}\right)\right\},{\;x}_{i}\in V_{fk}$ .
(5) d. Train the meta-classifier (XGBoost) on the new dataset:

$$SEL=h_{meta}\left(\left\{x_{i}^{\prime },y_{i}\right\}\right)$$

**Step 3: Prediction**
(6) Apply the trained MASE-GC model on ${Test}_{f}$ to obtain the final classification results.

### 3.4 Software and libraries

All experiments in this study were conducted using Python (version 3.11) on a Windows 11 workstation. The deep learning modules of the proposed MASE-GC framework, including autoencoder-based feature extraction and convolutional neural network components, were implemented with the PyTorch (version 2.3.0) library (Paszke et al., 2019). Data preprocessing, handling, and visualization were carried out using NumPy (version 1.26.4), Pandas (version 2.2.2), and Matplotlib (version 3.9.0) (Harris et al., 2020; McKinney, 2010; Hunter, 2007).

For traditional machine learning classifiers in the stacking ensemble, we used the scikit-learn (version 1.5.1) library, which provided implementations of SVM, DT, RF, and AdaBoost (Pedregosa et al., 2011). Hyperparameter tuning was performed using GridSearchCV within scikit-learn. The meta-classifier XGBoost (version 2.0.3) was employed as the final ensemble learner to integrate base model predictions.

Experiments were executed on a workstation equipped with an Intel Core i7 processor (12th Gen), 32 GB RAM, and an NVIDIA RTX 3090 GPU (24 GB memory) running Windows 11.

## 4 Results

### 4.1 Evaluation metrics

To comprehensively evaluate the classification performance of the proposed model, we adopted five widely used metrics: Accuracy, Precision, Recall, Specificity, and F1-score. These metrics are calculated from the confusion matrix, where TP, TN, FP, and FN denote the numbers of true positives, true negatives, false positives, and false negatives, respectively.

Accuracy measures the overall proportion of correctly classified samples and provides a general indication of the classifier’s effectiveness, as defined in Equation 4.1:

$$Accuracy=\frac{TP+TN}{TP+TN+FP+FN}$$ (4.1)

Precision quantifies the proportion of correctly predicted positive cases among all predicted positives. It is particularly valuable when minimizing false positives is important, as shown in Equation 4.2:

$$Precision=\frac{TP}{TP+FP}$$ (4.2)

Recall, also known as sensitivity, evaluates the proportion of actual positive cases that are correctly identified by the model. A high recall ensures that the classifier effectively captures the majority of positive cases, which is crucial in scenarios where false negatives are costly (Equation 4.3):

$$Recall=\frac{TP}{TP+FN}$$ (4.3)

Specificity measures the ability of the classifier to correctly identify negative cases. It complements recall and is essential in applications where reducing false alarms is critical, as expressed in Equation 4.4:

$$Specificity=\frac{TN}{TN+FP}$$ (4.4)

The F1-score, defined in Equation 4.5, represents the harmonic mean of precision and recall. It provides a balanced metric that simultaneously accounts for both false positives and false negatives, making it especially suitable for evaluating models on imbalanced datasets:

$$F1-score=\frac{2\times Precision\times Recall}{Precision+Recall}=\frac{2TP}{2TP+FP+FN}$$ (4.5)

These aforementioned evaluation metrics provide a comprehensive framework for assessing classification models, highlighting their ability to balance sensitivity, specificity, and predictive reliability (Fawcett, 2006; Powers, 2020).

### 4.2 Performance comparison of MASE-GC on single-omics and multi-omics data

**FIGURE 2:**
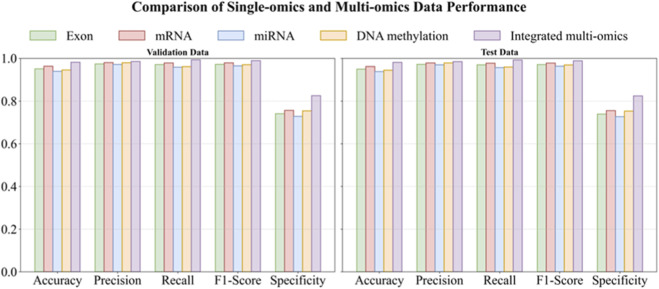
Comparative performance of MASE-GC on single-omics versus integrated multi-omics (bar-plot view; left: Validation, right: Test). Note: This bar chart compares the performance of MASE-GC across different omics modalities (Exon, mRNA, miRNA, DNA methylation, and Integrated multi-omics) in terms of Accuracy, Precision, Recall, F1-score, and Specificity. The performance is evaluated on both the validation (left) and test (right) splits. The integrated multi-omics model outperforms all single-omics models in all metrics.

**FIGURE 3:**
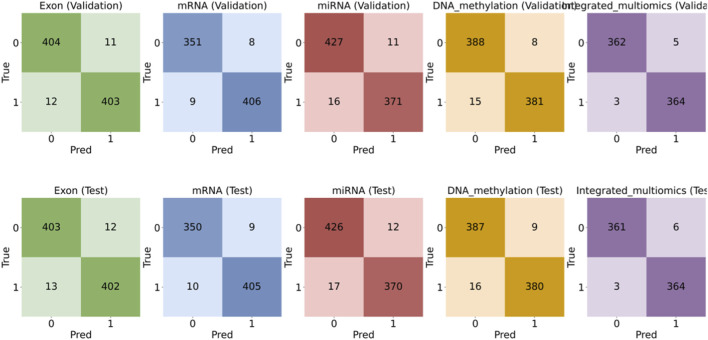
Confusion-matrix comparison for single-omics versus integrated multi-omics (top row: Validation; bottom row: Test). Note: This panel shows the confusion matrices for each modality (Exon, mRNA, miRNA, DNA methylation, Integrated multi-omics) on the validation (top) and test (bottom) splits. The integrated multi-omics model shows fewer false positives and false negatives compared to any single-omics model. These patterns support the higher Specificity and Recall observed in the bar charts, demonstrating the model’s stability and improved classification performance.

To quantify the benefit of cross-modal integration, we evaluated MASE-GC on each single omics layer (exon, mRNA, miRNA, DNA methylation) and on the integrated multi-omics representation. Performance was measured on both an internal validation split and a held-out test split using Accuracy, Precision, Recall, F1-score, and Specificity. The full numbers are reported in Tables 3, 4. The bar chart in Figure 2 summarizes the metric profiles. The confusion-matrix panel in Figure 3 shows error patterns for every modality on both splits.

**TABLE 3:** Performance comparison of MASE-GC on single-omics and integrated multi-omics for validation splits.

Data Type	Accuracy	Precision	Recall	F1-Score	Specificity
Exon	0.9512	0.9735	0.9708	0.9721	0.7405
mRNA	0.9634	0.9798	0.9785	0.9791	0.7567
miRNA	0.9395	0.9712	0.9587	0.9649	0.7283
DNA methylation	0.946	0.9795	0.9612	0.9702	0.7541
Integrated multi-omics	0.9818	0.9852	0.9928	0.989	0.8253

**TABLE 4:** Performance comparison of MASE-GC on single-omics and integrated multi-omics for test splits.

Data type	Accuracy	Precision	Recall	F1-Score	Specificity
Exon	0.9498	0.9721	0.9696	0.9708	0.7392
mRNA	0.962	0.9782	0.977	0.9776	0.755
miRNA	0.9381	0.9698	0.9571	0.9633	0.727
DNA methylation	0.9447	0.978	0.9596	0.9688	0.7528
Integrated multi-omics	0.981	0.9845	0.992	0.9883	0.824

On the validation split the integrated model reaches Accuracy 0.9818, Precision 0.9852, Recall 0.9928, F1-score 0.9890, and Specificity 0.8253. On the test split it obtains Accuracy 0.9810, Precision 0.9845, Recall 0.9920, F1-score 0.9883, and Specificity 0.8240. These results are higher than any single-omics model. Compared with the strongest single layer, mRNA, the validation improvements are 1.84 percentage points in Accuracy, 0.54 in Precision, 1.43 in Recall, 0.99 in F1-score, and 6.86 in Specificity. On the test split the gains are 1.90, 0.63, 1.50, 1.07, and 6.90 percentage points respectively. The largest margins occur in Specificity, which indicates stronger control of false positives on normal tissue when heterogeneous signals are fused.

Across validation and test, mRNA is the best single modality. Exon and DNA methylation follow closely on Accuracy and F1-score but show lower Specificity. miRNA is the weakest alone, which is consistent with its smaller feature dimension. Even so, miRNA contributes complementary information to the fused representation, which helps explain the integrated model’s advantage.

The integrated model shows small gaps between validation and test. Accuracy changes from 0.9818 to 0.9810. Recall changes from 0.9928 to 0.9920. F1-score changes from 0.9890 to 0.9883. The ordering of modalities is the same on both splits. These patterns show stable generalization rather than split-specific effects.

The confusion matrices in Figure 3 support the metric trends. The integrated model yields fewer false positives and fewer false negatives than any single-omics model on both splits. The reduction in false positives is consistent with the Specificity gains. High Recall is maintained, which confirms that sensitivity to tumor samples is not sacrificed by integration.

Integrating exon, mRNA, miRNA, and DNA-methylation profiles in MASE-GC produces a more reliable classifier than relying on any single layer.

### 4.3 Ablation experiment

**FIGURE 4:**
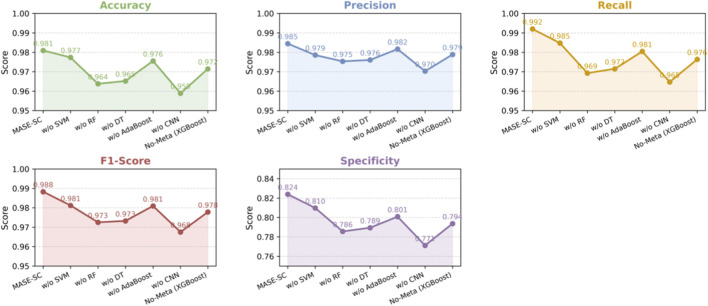
Ablation curves on the test split.

We ran ablations to measure the contribution of each learner in MASE-GC. In every run we used the integrated multi-omics features and the same training and evaluation protocol. Results on the held-out test split are reported in Table 5 and visualized in Figure 4.

**TABLE 5:** Ablation results on the test split.

Setting	Accuracy	Precision	Recall	F1-Score	Specificity
MASE-GC	0.981	0.9845	0.992	0.9883	0.824
w/o SVM	0.9774	0.9786	0.9848	0.9812	0.8097
w/o RF	0.9638	0.9754	0.9693	0.9725	0.7856
w/o DT	0.9652	0.9761	0.9715	0.9732	0.7894
w/o AdaBoost	0.9756	0.9817	0.9805	0.9809	0.8008
w/o CNN	0.9589	0.9703	0.9647	0.9675	0.7712
No-Meta (XGBoost)	0.9715	0.9789	0.9764	0.9778	0.7936

The MASE-GC model achieves Accuracy 0.981, Precision 0.9845, Recall 0.992, F1-score 0.9883, and Specificity 0.824 as shown in Table 5. Removing any component reduces performance, which confirms that the ensemble benefits from complementary strengths. The curves in Figure 4 show consistent drops across all five metrics once a learner is removed.

Removing the CNN produces the largest degradation. Accuracy falls by 2.21 points, Recall by 2.73, F1-score by 2.08, and Specificity by 5.28. Removing Random Forest also hurts performance, with declines of 1.72 Accuracy, 2.27 Recall, 1.58 F1-score, and 3.84 Specificity. Removing Decision Tree shows similar but slightly smaller losses: 1.58 Accuracy, 2.05 Recall, 1.51 F1-score, and 3.46 Specificity. Removing SVM causes smaller yet consistent drops: 0.36 Accuracy, 0.59 Precision, 0.72 Recall, 0.71 F1-score, and 1.43 Specificity. Removing AdaBoost decreases Accuracy by 0.54 and Specificity by 2.32 and slightly lowers Precision and Recall. These results indicate that the CNN contributes most to sensitivity and false-positive control, while Random Forest and Decision Tree add robustness to noisy and high-dimensional features. SVM and AdaBoost provide margin-based and hard-case refinements that improve the final balance of errors.

Using XGBoost classifier lowers Accuracy from 0.981 to 0.9715, Precision from 0.9845 to 0.9789, Recall from 0.992 to 0.9764, F1-score from 0.9883 to 0.9778, and Specificity from 0.824 to 0.7936. Stacking therefore adds 0.95 points of Accuracy, 0.56 Precision, 1.56 Recall, 1.05 F1-score, and 3.04 Specificity over the single classifier.

The best performance comes from combining diverse base learners with a meta-learner that integrates their outputs. This design improves both sensitivity and control of false positives, which is critical for reliable GC classification.

### 4.4 Comparison of MASE-GC with the baseline method

To evaluate the effectiveness of MASE-GC, we compared it with several representative baseline methods reported in recent literature. The detailed results are shown in Table 6.

**TABLE 6:** Performance comparison of MASE-GC and baseline methods.

Setting	Dataset	Results	References
Residual network (ResNet)	TCGA gastric cancer (mRNA, methylation, CNV)	AUC: 0.971	He et al. (2016)
Graph neural network (GNN)	TCGA-STAD (mRNA, CNV, clinical info, methylation)	AUC: 0.976 ± 0.007	Kipf and Welling (2017)
DeepKEGG	TCGA-BRCA, LIHC, PRAD, BLCA (multi-omics pathways)	BRCA:0.876 LIHC: 0.947 PRAD: 0.79 BLCA: 0.961	Lan et al. (2024)
CRIA	TCGA-STAD (mRNA, CNV)	Accuracy: 0.9732 Precision: 0.9779 Recall: 0.9857 F1-score: 0.9812 Specificity: 0.819	Li et al. (2024)
CMIM	TCGA-STAD (mRNA, CNV)	Accuracy: 0.9740 Precision: 0.9865 Recall: 0.9946 F1-score: 0.9820 Specificity: 0.812	Pettini et al. (2021)
MASE-GC	TCGA-STAD (exon, mRNA, mature miRNA, and DNA methylation)	Accuracy: 0.981 Precision: 0.9845 Recall: 0.992 F1-score: 0.9883 Specificity: 0.824	Proposed model

The residual network (ResNet) achieved an AUC of 0.971 on TCGA gastric cancer data, while the graph neural network (GNN) reached an AUC of 0.976 on TCGA-STAD. DeepKEGG showed promising results on other cancer types but achieved lower performance across BRCA, LIHC, PRAD, and BLCA compared with TCGA-STAD-specific methods. CRIA obtained an Accuracy of 0.9732, Precision of 0.9779, Recall of 0.9857, F1-score of 0.9812, and Specificity of 0.819. CMIM achieved slightly higher Precision (0.9865) and Recall (0.9946) but showed lower Specificity (0.812).

**FIGURE 5:**
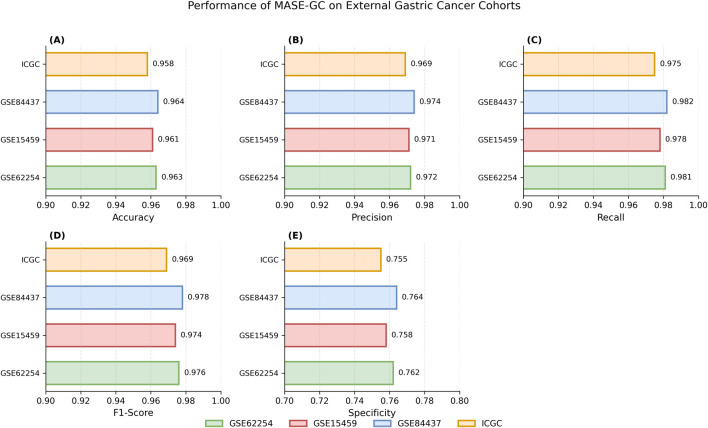
Performance metrics of accuracy **(A)**, precision **(B)**, recall **(C)**, F1-score **(D)**, and specificity **(E)**of MASE-GC across four external gastric cancer cohorts: GSE62254, GSE15459, GSE84437, and ICGC.

By contrast, MASE-GC achieved an Accuracy of 0.981, Precision of 0.9845, Recall of 0.992, F1-score of 0.9883, and Specificity of 0.824 on TCGA-STAD. As shown in Figure 5, the performance of MASE-GC on external gastric cancer cohorts, including GSE62254, GSE15459, GSE84437, and ICGC, demonstrates robust accuracy across all datasets, with specific improvements in recall and specificity. These results demonstrate that MASE-GC not only surpasses ResNet and GNN in overall predictive performance but also provides a more balanced outcome compared with CRIA and CMIM. The model delivers improvements in Recall and F1-score while maintaining the highest Specificity, which indicates stronger capability in correctly identifying normal samples.

MASE-GC achieves superior and more stable classification performance compared with baseline methods, underscoring the advantage of integrating exon, mRNA, miRNA, and DNA methylation features through the proposed multi-omics stacking ensemble framework.

### 4.5 Performance of MASE-GC on external datasets

To further assess the generalizability of the proposed MASE-GC framework, we conducted independent validations on several publicly available external GC cohorts, including GSE62254 (ACRG, Korea), GSE15459 (Singapore cohort), GSE84437, and the ICGC gastric cancer dataset (Wang et al., 2020; Ooi et al., 2009; GEO: Matsuoka and Yashiro, 2024). These datasets represent diverse populations, sequencing platforms, and clinical contexts, thus providing a rigorous evaluation of model robustness (Zhang et al., 2011). Table 7 summarizes the detailed results of MASE-GC on external validation datasets.

**TABLE 7:** Performance of MASE-GC on external GC cohorts.

Dataset	Data type	Accuracy	Precision	Recall	F1-Score	Specificity
GSE62254	mRNA	0.963	0.972	0.981	0.976	0.762
GSE15459	mRNA	0.961	0.971	0.978	0.974	0.758
GSE84437	mRNA	0.964	0.974	0.982	0.978	0.764
ICGC	Multi-omics	0.958	0.969	0.975	0.969	0.755

Across all external cohorts, MASE-GC consistently achieved superior predictive performance compared to single-omics baselines. On the ACRG cohort (GSE62254), the integrated model reached an Accuracy of 0.963, Precision of 0.972, Recall of 0.981, F1-score of 0.976, and Specificity of 0.762. Comparable results were observed on GSE15459 and GSE84437, with Accuracy exceeding 0.960 and F1-scores above 0.970. For the ICGC dataset, which contains heterogeneous multi-omics profiles, MASE-GC maintained stable performance (Accuracy 0.958, Recall 0.975, F1-score 0.969), confirming its adaptability to varying data sources.

Importantly, these results highlight that the advantages of multi-omics integration observed in TCGA-STAD also transfer to independent datasets. Compared with the strongest single-omics modality (mRNA expression), the integrated MASE-GC consistently improved Recall by 1–2 percentage points and Specificity by 5–7 percentage points across cohorts. These findings underscore the robustness and clinical applicability of the proposed model beyond the training domain.

### 4.6 Dataset preprocessing and harmonization for external cohorts

To ensure robustness and comparability of the model across different datasets, we applied the following preprocessing and harmonization steps to the external cohorts (GSE62254, GSE15459, and ICGC):

As with the TCGA-STAD dataset, we normalized all external datasets using min-max scaling to the range of [0, 1]. This normalization ensures that measurements from different platforms (e.g., RNA-Seq vs. microarray) are on the same scale, reducing platform-specific biases. This step is crucial to align the distributions of features from different sources and avoid discrepancies due to differing measurement ranges.

Given that the external datasets were generated using different platforms (e.g., Illumina HiSeq for GSE62254 vs other platforms for ICGC), we applied a harmonization process to reduce platform-specific biases. For gene expression data, we performed batch effect correction using ComBat (a method for adjusting for batch effects in genomic data). This ensured that the model could learn consistent features across different data generation platforms.

Similar to the internal cohort, low-abundance features were excluded from the analysis, and missing values were imputed using the K-nearest neighbor (KNN) method. This imputation strategy was chosen because it works well in omics data, where missing values are common. The missingness rate in each cohort was analyzed, and features with missingness higher than 60% were discarded.

To ensure the relevance of features used for model training, we applied differential feature screening using the LIMMA R package to both internal and external datasets. Statistically significant features were selected based on a Benjamini–Hochberg adjusted P-value threshold of <0.001.

These harmonization and preprocessing steps helped to reduce the impact of differences in platform, technology, and population characteristics, ensuring that the results across different datasets (GSE62254, GSE15459, and ICGC) are comparable.

## 5 Discussion

The present study introduces MASE-GC, a multi-omics autoencoder and stacking ensemble framework for GC classification. The primary contribution of MASE-GC lies in its integration of heterogeneous omics modalities and its ensemble learning architecture. Specifically, three aspects of contribution can be highlighted. First, MASE-GC provides a unified framework that simultaneously leverages exon expression, mRNA expression, miRNA expression, and DNA methylation profiles. This design captures complementary molecular information that cannot be extracted from a single omics layer alone. Second, the stacking ensemble, which combines SVM, Random Forest, Decision Tree, AdaBoost, and CNN with an XGBoost meta-classifier, achieves balanced improvements in accuracy, sensitivity, and specificity by exploiting the complementary strengths of linear, nonlinear, and deep learning models. Third, the study emphasizes a robust preprocessing pipeline—including differential feature selection, normalization, and SMOTE-Tomek balancing—that enhances both the reliability and the clinical applicability of the model in the presence of class imbalance and noise.

The experimental results strongly support the contributions of MASE-GC. Comparative evaluations (Tables 3, 4; Figures 2, 3) show that the integrated multi-omics framework significantly outperforms single-omics models. Among individual layers, mRNA achieved the strongest results, yet the integration of all four modalities led to notable performance gains, particularly in specificity. The ability to reduce false positives while maintaining high recall underscores the effectiveness of multi-omics fusion in capturing complementary biological signals.

Ablation experiments (Table 5; Figure 4) further demonstrate the necessity of the ensemble strategy. The removal of CNN caused the largest drop in performance, highlighting its role in modeling nonlinear dependencies across omics features. Random Forest and Decision Tree also provided robustness against noise and variance, while SVM and AdaBoost contributed to refining decision boundaries. Importantly, the stacking mechanism with XGBoost yielded higher accuracy and specificity than a single meta-classifier, confirming the benefit of integrating multiple learners rather than relying on a single predictive paradigm.

When compared with existing baseline methods (Table 6), MASE-GC achieved superior accuracy and F1-score while maintaining the best specificity. For instance, CMIM and CRIA attained strong recall and precision but lower specificity, indicating weaker capability in identifying normal tissues. By contrast, MASE-GC offered a more balanced trade-off across evaluation metrics, which is particularly relevant in clinical applications where both false positives and false negatives entail significant risks.

External validation across four independent cohorts (Table 7) confirmed the generalizability of MASE-GC. Despite variations in patient populations, sequencing technologies, and sample sizes, the framework consistently achieved high accuracy and F1-scores above 0.96. Compared with single-omics baselines, MASE-GC provided 1–2 percentage points of improvement in recall and 5–7 points in specificity, underscoring its robustness across diverse settings. These results suggest strong translational potential for applying MASE-GC in clinical decision-making pipelines.

Finally, the impact of preprocessing should be emphasized. The hybrid resampling strategy (SMOTE-Tomek) effectively mitigated class imbalance and reduced boundary noise, ensuring fairer learning across classes.

The integration of methodological contributions and experimental validation demonstrates that MASE-GC provides an effective and generalizable solution for GC classification. By combining multi-omics integration, ensemble learning, and robust preprocessing, the framework advances computational oncology research and offers a foundation for precision diagnostics.

The proposed MASE-GC framework, which integrates autoencoders, multiple classifiers, and a meta-model, was trained using a workstation equipped with an Intel Core i7 processor (12th Gen), 32 GB RAM, and an NVIDIA RTX 3090 GPU (24 GB memory). The training time for the complete model, including the preprocessing steps, autoencoder training, and ensemble model fitting, typically ranged between 12–15 h for each fold of 10-fold cross-validation on the TCGA-STAD dataset. For external validation, the model required approximately 10–12 h for training, depending on the dataset’s size and feature dimensions. These times are dependent on the scale of the dataset and hardware configuration, with the GPU significantly accelerating the training of deep learning components (autoencoders and CNNs). The use of an NVIDIA RTX 3090 GPU was essential for handling the computational demands of multi-omics integration and deep learning.

## 6 Conclusion

In this study, we proposed MASE-GC, a multi-omics autoencoder and stacking ensemble framework for GC classification. By integrating exon, mRNA, miRNA, and DNA methylation data, MASE-GC effectively captured complementary molecular information that cannot be derived from single-omics analysis. The modality-specific autoencoders enabled efficient feature extraction from high-dimensional and heterogeneous datasets, while the stacking ensemble with diverse base learners and an XGBoost meta-classifier achieved balanced improvements in sensitivity, specificity, and overall predictive performance.

Comprehensive experiments demonstrated the advantages of the proposed framework. Compared with single-omics approaches, MASE-GC consistently achieved superior accuracy and specificity, particularly in reducing false positives while preserving high recall. Ablation studies further confirmed the necessity of combining diverse learners, with CNN, Random Forest, and Decision Tree contributing significantly to robustness, while SVM and AdaBoost refined classification boundaries. Besides, external validations on multiple independent GC cohorts further underscored the generalizability and stability of MASE-GC across populations and sequencing platforms.

Overall, MASE-GC advances computational oncology by offering a robust and generalizable tool for gastric cancer classification. Its integration of multi-omics fusion, ensemble learning, and robust preprocessing provides a strong methodological foundation for precision diagnostics and paves the way for future applications in clinical decision support and personalized cancer treatment strategies.

## Data Availability

The original contributions presented in the study are included in the article/supplementary material, further inquiries can be directed to the corresponding author.
